# An Assessment of PI-RADS v2.1: Inter-Reader Agreement, Diagnostic Accuracy, and the Added Value of PSA Density in Prostate Cancer Prediction

**DOI:** 10.3390/diagnostics16183056

**Published:** 2026-09-20

**Authors:** Mohammad Abufaraj, Sarah B. Al-Qalalweh, Ahmad S. Kreishan, Rama M. Abbadi, Omar Ismail, Abdelrhman Labib, Mohammad Abu Shattal, Diana A. Bawi, Osama Samara, Yazeed E. Alhanbali, Salma N. Farah, Mousa A. Al-Abbadi, Shahrokh F. Shariat

**Affiliations:** 1 Division of Urology, Department of Special Surgery, Jordan University Hospital, The University of Jordan, Amman 11942, Jordan; sfshariat@gmail.com; 2 Department of Urology, Comprehensive Cancer Center Vienna, Medical University of Vienna, 1090 Vienna, Austria; 3 Department of Special Surgery, Jordan University Hospital, University of Jordan, Amman 11942, Jordan; sarabasel258@gmail.com; 4 Department of Radiology, King Hussein Medical Center, Jordanian Royal Medical Services, Amman 11733, Jordan; dr.kreishan@gmail.com; 5 Department of Psychiatry and Behavioral Sciences, University of Minnesota, Minneapolis, MN 55454, USA; ramaabbadyy@gmail.com; 6 Department of Internal Medicine, Jordan University Hospital, University of Jordan, Amman 11942, Jordan; omar.moh.isml@gmail.com; 7 Department of Radiology, Abdali Hospital, Amman 11191, Jordan; abdelrhman.labib@gmail.com; 8 Department of Diagnostic Radiology, King Hussein Cancer Center, Amman 11941, Jordan; ma.11707@khcc.jo; 9 University Hospitals Leicester NHS Trust, Leicester LE1 5WW, UK; dianasuhbat@gmail.com; 10 Department of Radiology and Nuclear Medicine, Jordan University Hospital, The University of Jordan, Amman 11942, Jordan; osamara@ju.edu.jo; 11 Department of Internal Medicine, The University of Texas Medical Branch, Galveston, TX 77555, USA; yealhanb@utmb.edu; 12 School of Medicine, University of Jordan, Amman 11942, Jordan; salmafarah2002@hotmail.com; 13 Department of Pathology, Microbiology and Forensic Medicine, College of Medicine, University of Jordan, Amman 11942, Jordan; ma.alabbadi@ju.edu.jo; 14 Department of Urology, Weill Cornell Medical College, New York, NY 10065, USA

**Keywords:** prostate cancer, PI-RADS v2.1, multiparametric MRI, inter-reader agreement, PSA density, clinically significant prostate cancer

## Abstract

**Introduction:** Multiparametric MRI (mpMRI) interpreted using Prostate Imaging Reporting and Data System (PI-RADS) v2.1 is central to prostate cancer (PCa) assessment, but inter-reader variability remains a concern. This study evaluated inter-reader agreement, diagnostic performance, the utility of prostate-specific antigen density (PSA-D), and imaging predictors of biopsy-detected PCa. **Methods:** We conducted a retrospective study of 102 patients who underwent prostate mpMRI followed by transrectal ultrasound (TRUS)-guided biopsy. Three experienced radiologists independently scored examinations using PI-RADS v2.1. Agreement was assessed using intraclass correlation coefficients (ICC) and weighted κ statistics. Diagnostic performance was evaluated at a PI-RADS ≥3 threshold. Patients were stratified by PSA-D, and logistic regression identified imaging predictors of biopsy outcomes. Reader confidence was scored on a 10-point scale. **Results:** PCa was detected in 60/102 patients (58.8%) and csPCa in 46/102 (45.1%). Overall agreement was moderate to good (ICC, 0.749; κ, 0.748), with higher agreement in the transitional compared to the peripheral zone (ICC, 0.822 vs. 0.662). Averaged PI-RADS scores yielded 100% sensitivity for both PCa and csPCa, with specificities of 47.6% and 35.7%, respectively. PSA-D stratification showed higher PPVs at PSA-D ≥0.15 ng/mL^2^, while sensitivity and NPV were 100% in both strata; specificity remained relatively low. Reader confidence correlated with PI-RADS category and interpretation accuracy. In multivariable patient-level analyses, DWI hyperintensity was independently associated with biopsy-detected PCa and csPCa. **Conclusions:** PI-RADS v2.1 showed moderate to good inter-reader agreement and high sensitivity but modest specificity for PCa and csPCa. Agreement was higher for transitional-zone lesions, and DWI hyperintensity was independently associated with PCa and csPCa. PSA-D subgroup estimates were imprecise, and its incremental value requires confirmation in larger prospective cohorts.

## 1. Introduction

Prostate cancer (PCa) is the most commonly diagnosed malignancy among men in the United States [1]. Traditionally, diagnosis relies on PSA measurement, digital rectal examination, and 12-core ultrasound-guided biopsy, an approach prone to both overdiagnosis and underdiagnosis [2,3]. Multiparametric MRI (mpMRI) has substantially improved diagnostic precision, enabling tumor localization, refinement of active surveillance decisions, and a reduction in unnecessary biopsies through targeted biopsy techniques [4,5].

A major barrier to broader mpMRI adoption is variability in interpretation and reporting. Without standardization, mpMRI reports vary in terminology, content, and diagnostic thresholds, limiting reproducibility and clinical utility [6]. The Prostate Imaging-Reporting and Data System (PI-RADS) was developed to address this issue by providing a structured scoring framework. Since its initial release in 2012, PI-RADS has undergone iterative refinement; the current version, v2.1 (2019), introduced more objective criteria for transition zone lesion assessment and clarified technical specifications [7,8]. Despite these improvements, inter-reader variability remains a recognized limitation among readers with varying experience levels [9,10].

Beyond PI-RADS scoring, PSA-D, has emerged as a meaningful adjunct to mpMRI interpretation. PSA-D reflects tumor burden relative to gland size, correlates with Gleason grade and disease aggressiveness, and is particularly useful for refining the predictive value of intermediate PI-RADS scores where cancer risk is inherently ambiguous [11,12]. Incorporating PSA-D into risk stratification has been shown to reduce unnecessary biopsies while preserving detection of clinically significant PCa (csPCa) [13].

This study assesses the diagnostic accuracy and inter-reader variability of PI-RADS v2.1, explores diagnostic performance across PSA-D strata, and identifies mpMRI features associated with PCa detection.

## 2. Materials and Methods

**Figure 1:**
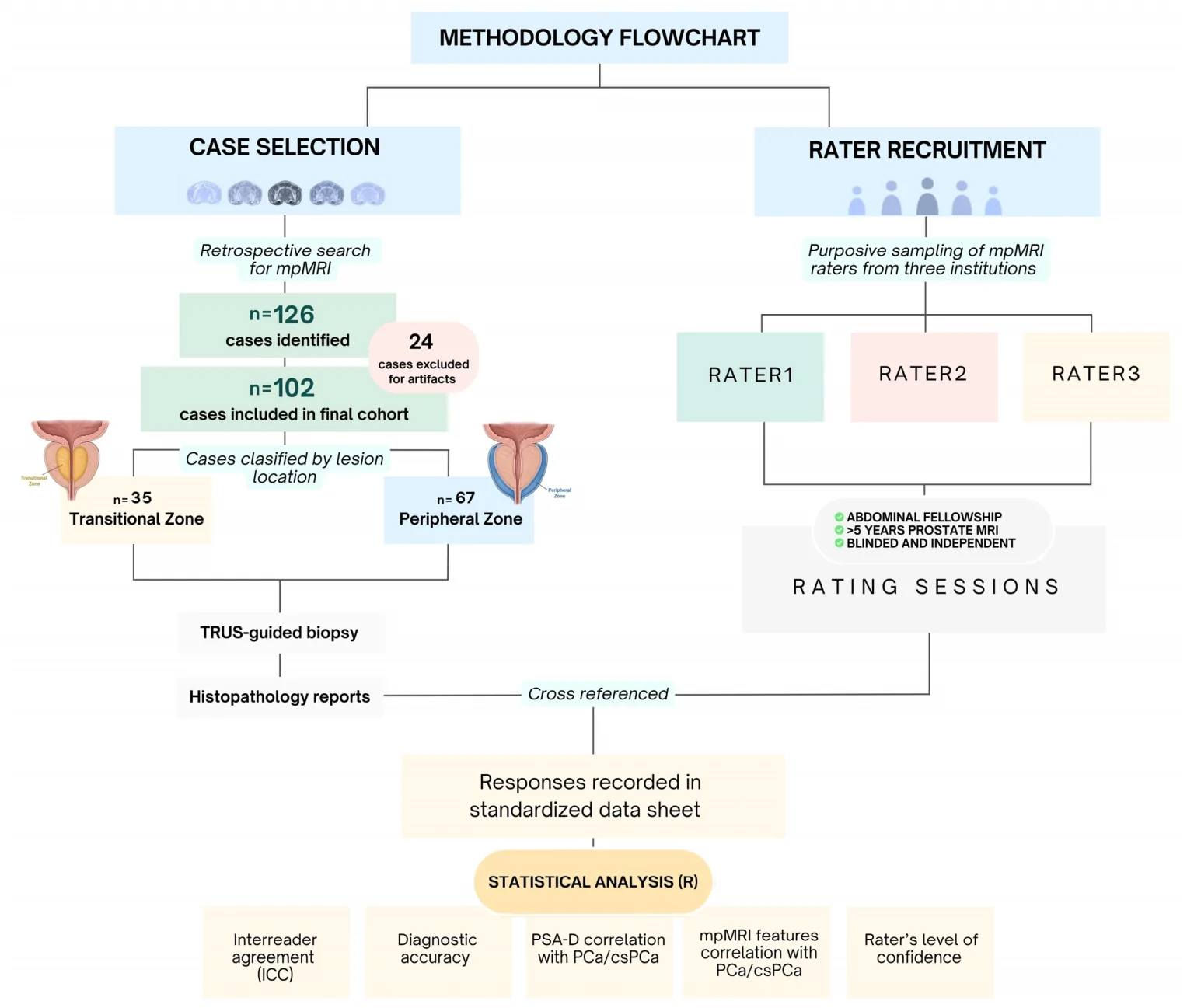
Overview of the study methodology.

The study design and protocol were approved by the Institutional Review Board (No. 10/2025/5463) and the Scientific Committee at Jordan University Hospital. The study was conducted in accordance with the Declaration of Helsinki. The requirement for informed consent was waived given the retrospective design. An overview of the methodology is summarized in Figure 1.

### 2.1. Image Selection and Preparation

A retrospective search of the institutional database identified prostate mpMRI examinations performed between 1 May 2019, and 12 December 2024. Only patients with corresponding TRUS-guided prostate biopsy results were included. The initial search yielded 126 cases; 24 were excluded due to significant motion artifacts or technical errors compromising image quality or lesion assessment, yielding a final cohort of 102 cases.

All mpMRI examinations were performed on a 3-Tesla MRI system (Siemens Verio, Siemens Healthineers, Erlangen, Germany). The protocol included T2-weighted imaging (T2WI) in sagittal, coronal, and axial planes; diffusion-weighted imaging (DWI) with b-values of 100, 600, and 1000 s/mm^2^, with a computed high b-value of 1600 s/mm^2^ and corresponding apparent diffusion coefficient (ADC) maps; and axial T1-weighted imaging obtained both before contrast administration and dynamically after contrast (DCE) with 20 temporal phases. Imaging was performed in accordance with PI-RADS technical recommendations [7].

### 2.2. Rater Selection

Three experienced mpMRI raters from three different institutions were recruited via purposive sampling. All had completed fellowships in abdominal imaging and had more than five years of dedicated prostate MRI experience.

### 2.3. Rating Sessions

Each rater independently evaluated all cases in structured, supervised sessions. Raters scored the most suspicious index lesion per PI-RADS v2.1 criteria and were provided with the official PI-RADS v2.1 instructional atlas. All sessions were conducted in a controlled environment using a shared software platform, and responses were recorded by a supervising author in a standardized data collection sheet.

For each case, raters recorded: PSA-D (calculated from provided PSA and prostate volume measured on mpMRI); index lesion location using a 41-sector map (38 prostatic sectors, 2 seminal vesicle sectors, 1 membranous urethra sector); lesion morphology and margins; features of extraprostatic extension (neurovascular bundle invasion, pelvic sidewall and bladder neck involvement); index lesion size; and signal characteristics on DWI, ADC maps, T2WI, and DCE imaging. Raters also indicated whether biopsy was warranted and rated their confidence in each response on a 10-point scale (1 = not confident; 10 = very confident). Clinical information provided to raters included indication for biopsy, serum PSA level, and prior biopsy history. Raters were blinded to each other’s assessments and to all prior radiological and histopathological findings. Supplementary Figures S1–S4 show a sample of four cases with complete concordance and complete discordance between the raters along with their corresponding biopsy results.

### 2.4. Biopsy

All patients underwent 12-core systematic TRUS-guided biopsy supplemented by 2–4 cognitively targeted cores directed to the index lesion identified on pre-biopsy mpMRI.

### 2.5. Histopathology

Biopsy specimens were graded according to ISUP 2014 recommendations [14]. Per institutional protocol, all reports represent the consensus of a resident pathologist who initially assessed the specimen and drafted the report, and an attending pathologist who reviewed the specimen and issued the final report. Cases with grading discordance between the study pathologist and the original report were adjudicated by an independent pathologist, and a consensus Gleason score was assigned.

### 2.6. Statistical Analysis and Sample Size

All analyses were performed using R [15] (irr, kappaSize, EpiR, multipleROC, and car packages). Continuous variables were presented as medians and interquartile ranges (IQR); categorical variables as frequencies and percentages. Statistical significance was defined as *p* < 0.05.

Inter-reader agreement was assessed using a two-way random effects intraclass correlation coefficient (ICC (2,1)) for both consistency and absolute agreement for single raters, and Conger’s generalized Kappa coefficient with quadratic weighting, both reported with their respective 95% confidence intervals (CI). The ICC (2,1) model was chosen because it assesses agreement and reliability among multiple raters using a scaled rating system while accounting for the magnitude of disagreement between ratings rather than treating all disagreements equally. Conger’s weighted kappa was additionally calculated to provide a complementary measure of agreement and to facilitate comparison with previous studies reporting kappa statistics. Agreement was assessed at the patient level, with one PI-RADS score per patient corresponding to the most suspicious index lesion.

Diagnostic performance was assessed at the patient level using sensitivity, specificity, and AUC at a PI-RADS ≥ 3 threshold. A patient was considered positive for PCa or csPCa (defined as grade group ≥ 2; Gleason score ≥ 3 + 4) if cancer was detected in any biopsy core, irrespective of core location relative to the reader-selected index lesion. Lesion-level radiologic-pathologic matching was not used for the diagnostic accuracy analyses. Associations between patient-level mpMRI features and biopsy outcomes were evaluated using univariate logistic regression. DWI, ADC, T2WI, and DCE findings recorded independently by the three raters were reduced to a single binary value per patient using majority voting. Features significant at *p* < 0.05 in univariate analyses were entered into a multivariable model. Results are reported as log odds ratios with 95% CIs.

Sample size was estimated based on ICC benchmarks proposed by Koo and Li [16], which define ICC 0.5–0.75 as moderate agreement. A sample of 97 was required to detect an ICC exceeding 0.5 with 80% power at a two-sided significance level of 0.05.

## 3. Results

A total of 102 patients who underwent multiparametric MRI (mpMRI) followed by transrectal ultrasound-guided prostate biopsy were included in the analysis. Baseline characteristics are summarized in Table 1. The median age was 66 years with median total PSA level of 10.54 (7.03–22.33) ng/mL. The median prostate volume was 58 (41.75–81.75) mL with a median PSA-D of 0.17 (0.1–0.43) ng/mL^2^. Sixteen patients (15.7%) were receiving finasteride.

On histopathological evaluation of TRUS-guided biopsy specimens, prostate adenocarcinoma was identified in 60 patients (58.8%). Benign prostatic hyperplasia was present in 14 patients (13.7%), prostatitis in 6 (5.9%), and prostatic intraepithelial neoplasia in 4 (3.9%). Among patients with adenocarcinoma, grade group 2 was the most frequent category, observed in 16 cases (26.7%), followed by grade group 1 in 14 cases (23.3%). Table 1 summarizes the histopathological features of the study population.

### 3.1. Inter-Reader Agreement

Table 2 shows the PI-RADS results for the three raters. The median PI-RADS score was 4 for all raters, with the majority of the sample being labeled as PI-RADS 4 or 5 by the three raters.

Inter-reader agreement among the three radiologists was moderate to good (ICC = 0.749 [95% CI, 0.672–0.814], *p* < 0.001, κ = 0.748 [0.669–0.827], *p* < 0.001). Agreement was higher for lesions in the transitional zone (ICC = 0.822 [0.704–0.904], *p* < 0.001, κ = 0.771 [0.643–0.899], *p* < 0.001) as compared with those in the peripheral zone (ICC = 0.662, [0.545–0.763], *p* < 0.001, κ = 0.676 [0.561–0.791], *p* < 0.001).

### 3.2. Diagnostic Accuracy

**Figure 2:**
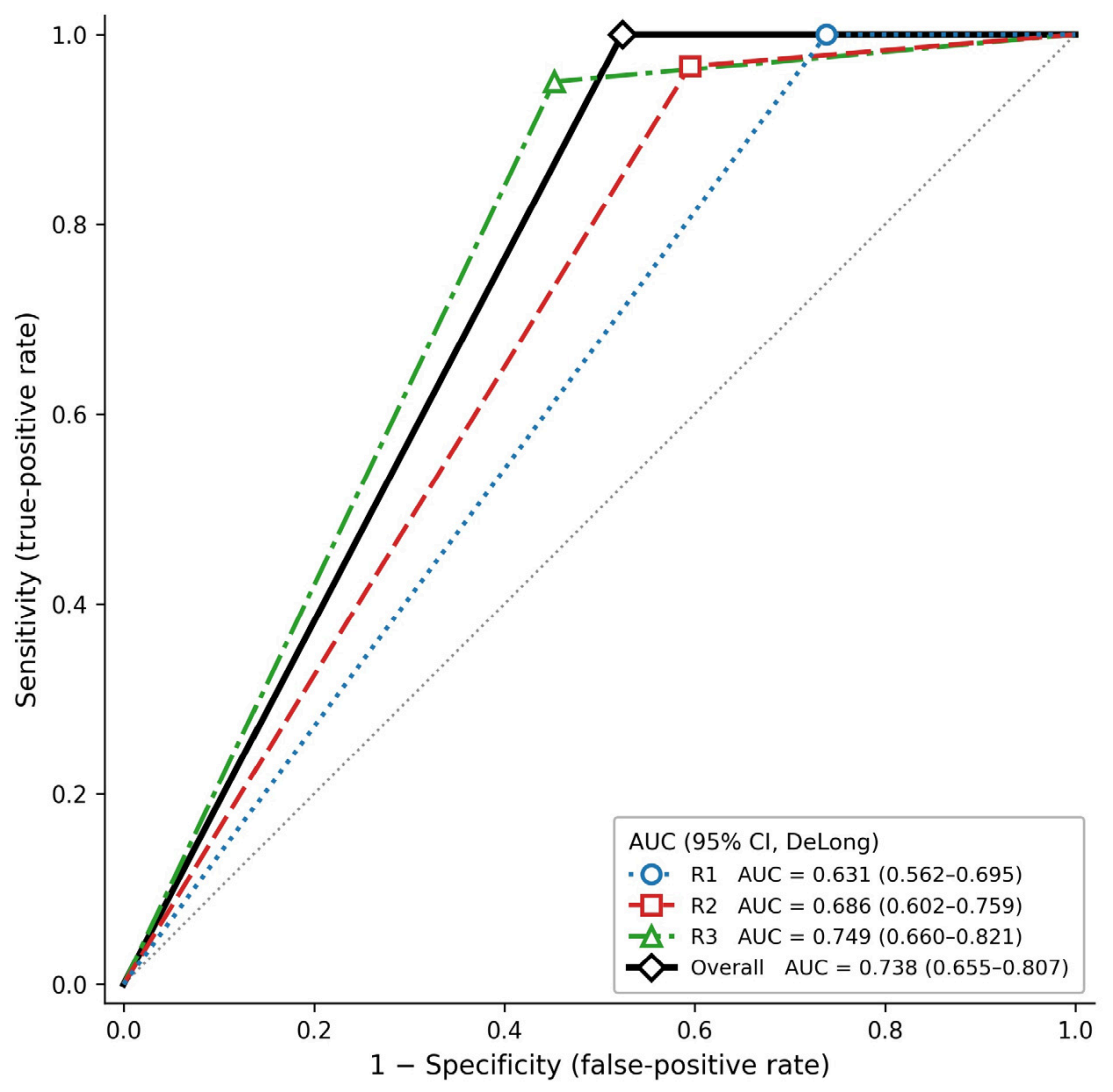
Receiver operating characteristic (ROC) curves for detection of any prostate cancer (PCa) using PI-RADS v2.1 by individual raters and the averaged score. Markers denote each rater’s single operating point; the diagonal dotted line indicates chance performance. Areas under the curve (AUC) with 95% confidence intervals are given in the legend.

**Figure 3:**
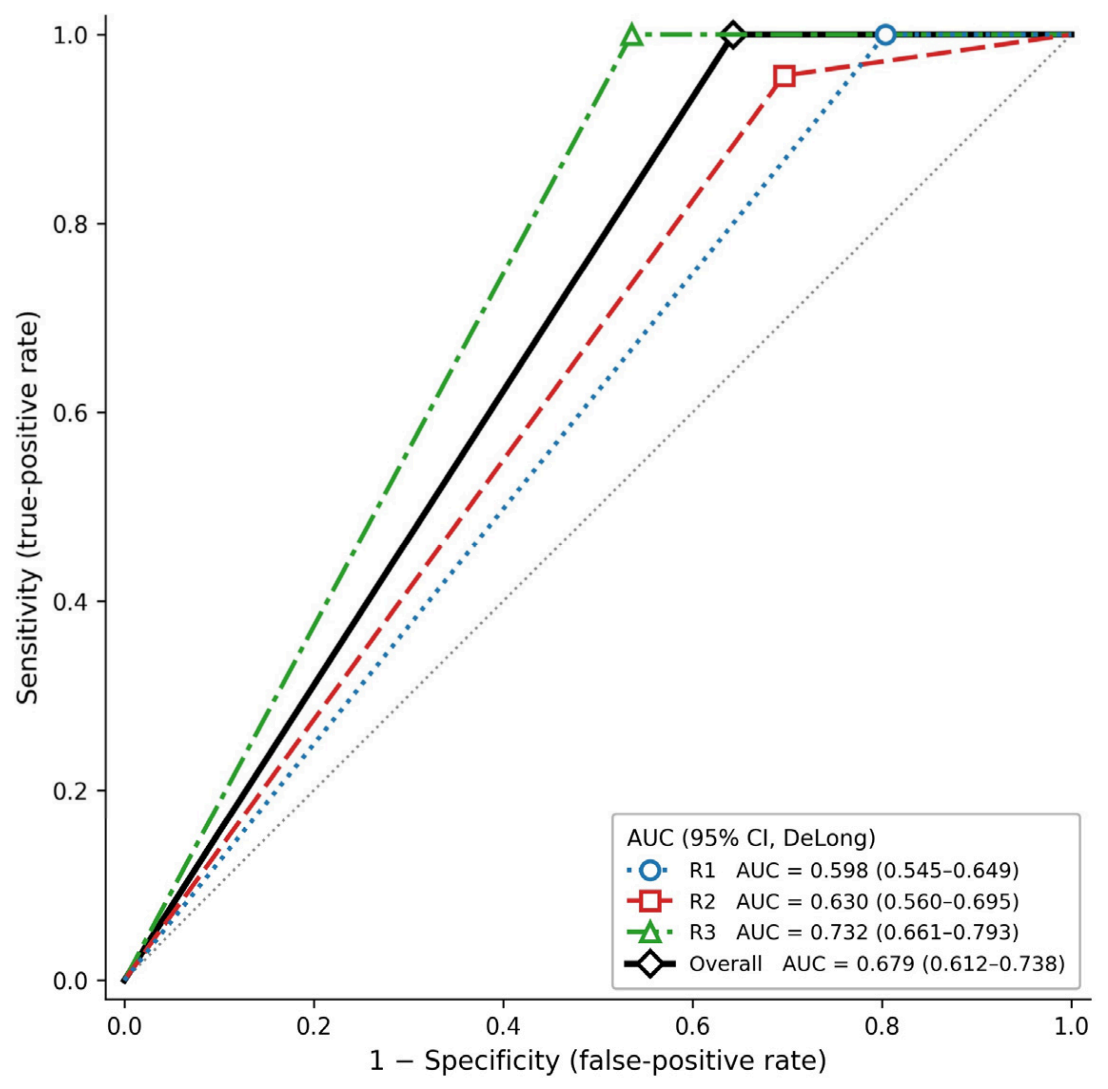
Receiver operating characteristic (ROC) curves for detection of clinically significant prostate cancer (csPCa) using PI-RADS v2.1 by individual raters and the averaged score. Markers denote each rater’s single operating point; the diagonal dotted line indicates chance performance. Areas under the curve (AUC) with 95% confidence intervals are given in the legend.

Table 3 shows the diagnostic performance of PI-RADS version 2.1 for PCa and csPCa. For detection of any PCa, the sensitivity was 100% for rater 1, 96.7% for rater 2, and 95% for rater 3 at the overall level, while the corresponding values for specificity were 26.2%, 40.5%, and 54.8%, respectively. For detection of csPCa, the overall sensitivity was 100% for rater 1, 95.7% for rater 2, and 100% for rater 3, with corresponding specificity of 19.6%, 30.4%, and 46.4%, respectively. When the average score across the three raters was considered, the overall sensitivity was 100% for both any PCa and csPCa, while the specificity was 47.6% and 35.7%, respectively. For detection of any PCa, the AUC was 0.631 for rater 1, 0.686 for rater 2, 0.749 for rater 3, and 0.738 for the average score across the three raters. For detection of csPCa, the AUC was 0.598 for rater 1, 0.630 for rater 2, 0.732 for rater 3, and 0.679 for the average score across the three raters. The corresponding ROC curves are shown in Figure 2 and Figure 3, respectively.

Zone-specific analysis showed that sensitivity was uniformly high in both zones (86.7–100% for any cancer and 85.7–100% for csPCa), whereas specificity was consistently higher in the transitional than the peripheral zone for every rater (52.6–73.7% versus 4.3–39.1% for any cancer and 50.0–70.0% versus 2.8–33.3% for csPCa).

### 3.3. PSA Density Effect

When patients were stratified by PSA-D, the higher PSA-D group demonstrated markedly higher PPVs for both PCa and csPCa, whereas sensitivity and NPV remained 100% in both groups. For the detection of any PCa, a PI-RADS score ≥3 yielded a sensitivity, specificity, NPV, and PPV of 100.0% (95% CI, 75.3–100.0%), 51.6% (95% CI, 33.1–69.8%), 100.0% (95% CI, 79.4–100.0%), and 46.4% (95% CI, 27.5–66.1%), respectively, in patients with PSA-D < 0.15 ng/mL^2^. The corresponding values in patients with PSA-D ≥ 0.15 ng/mL^2^ were 100.0% (95% CI, 92.5–100.0%), 36.4% (95% CI, 10.9–69.2%), 100.0% (95% CI, 39.8–100.0%), and 87.0% (95% CI, 75.1–94.6%), respectively.

For csPCa, sensitivity, specificity, NPV, and PPV were 100.0% (95% CI, 47.8–100.0%), 41.0% (95% CI, 25.6–57.9%), 100.0% (95% CI, 79.4–100.0%), and 17.9% (95% CI, 6.1–36.9%), respectively, in the low PSA-D subgroup, compared with 100.0% (95% CI, 91.4–100.0%), 23.5% (95% CI, 6.8–49.9%), 100.0% (95% CI, 39.8–100.0%), and 75.9% (95% CI, 62.4–86.5%), respectively, in the high PSA-D subgroup. The substantially higher PPVs in the high PSA-D subgroup occurred in the context of markedly higher disease prevalence, with PCa and csPCa prevalences of 81.0% and 70.7%, respectively, compared with 29.5% and 11.4% in the low PSA-D subgroup. Table 4 presents the results of the PSA-D stratification. Supplementary Table S1 specifically reports the PSA-D-stratified outcomes for PI-RADS 3 lesions.

### 3.4. Radiological Features on mpMRI

Radiologic abnormalities on mpMRI among the cohort are presented in Supplementary Table S2. DWI hyperintensity was present in 89 (87.3%) patients, ADC hypointensity in 93 (91.2%), hyperenhancement in 96 (94.1%), and T2 hypointensity in 100 (98.0%). All four radiologic features were observed in 100% of patients with PCa, whereas these findings were less frequent among patients without PCa (69.0%, 78.6%, 85.7%, and 95.2%, respectively).

The distribution of radiological findings showed quasi-complete separation, as the presence of each radiological feature was restricted to patients with PCa. Therefore, Firth’s penalized logistic regression was used. In univariate patient-level analyses, DWI hyperintensity, ADC hypointensity, and hyperenhancement of the reader-selected index lesion were significantly associated with biopsy-detected PCa and csPCa, whereas T2 hypointensity was not. In the multivariable patient-level analysis, DWI hyperintensity remained significantly associated with biopsy-detected PCa and csPCa, whereas ADC hypointensity and hyperenhancement were no longer statistically significant. T2 hypointensity was not included in the multivariable model because it did not meet the threshold for inclusion in the univariate analysis. Table 5 presents the patient-level associations between radiological features of the reader-selected index lesion and biopsy-detected PCa and csPCa.

### 3.5. Raters’ Level of Confidence

Self-reported confidence in PI-RADS ratings was consistently high across all three readers. The median confidence scores were 8 (6–10), 8 (7–9), and 8 (7–9) for the three raters, respectively. The overall average confidence across the three raters was 8 (7–9).

**Figure 4:**
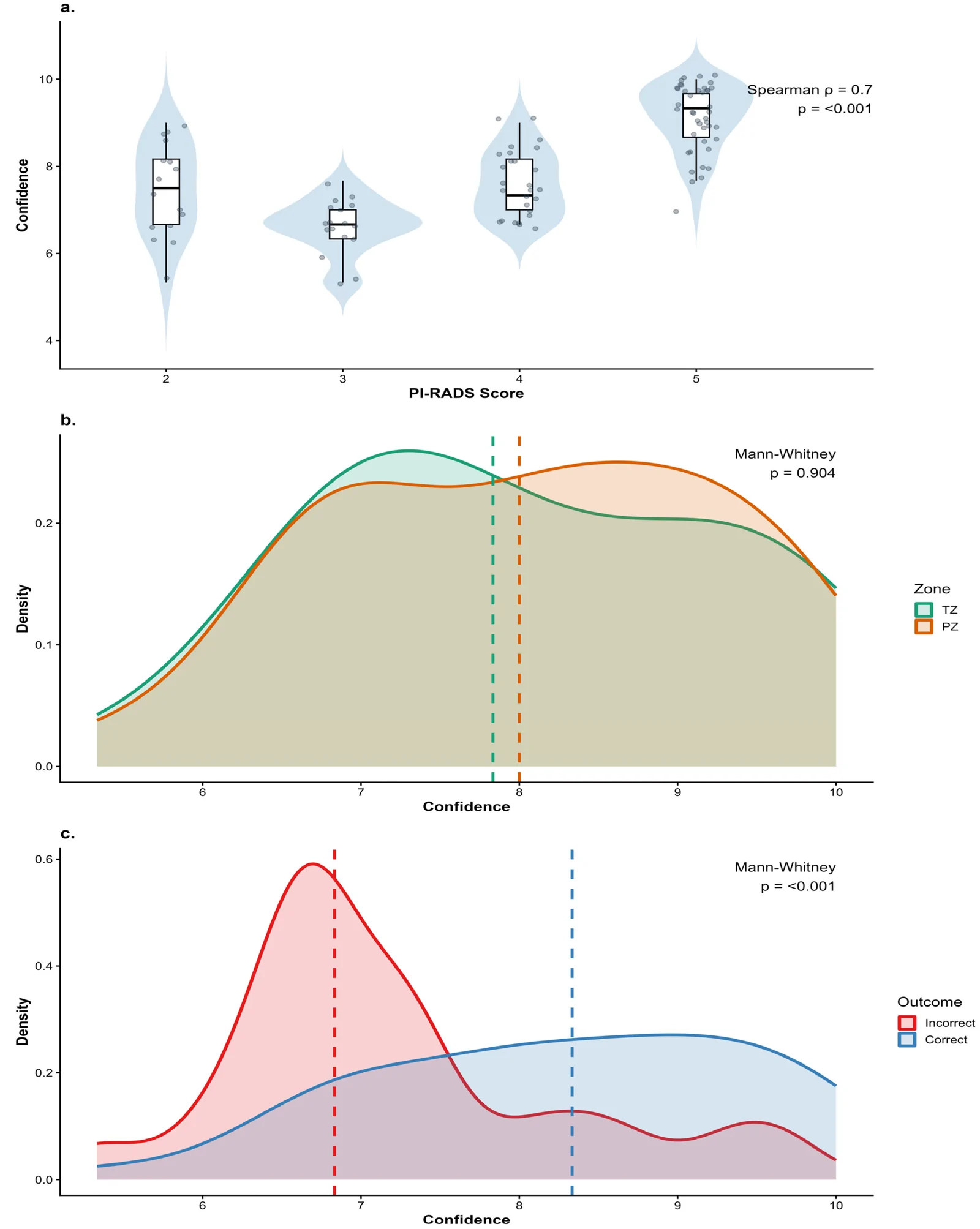
(**a**) Shows the distribution of the raters’ confidence for each PI-RADS category and the correlation between the two variables, (**b**) shows the difference in the raters’ confidence when assessing the two zones, and (**c**) shows the difference in the raters’ confidence between the rating that correctly predicted the presence or absence of prostate cancer compared to the ratings that incorrectly predicted them, TZ: Transitional zone, PZ: Peripheral zone.

Figure 4a shows the correlation between the level of confidence and the PI-RADS ratings. The raters’ confidence showed positive correlation with the PI-RADS ratings (Rho = 0.7, *p* < 0.001), where the confidence was higher in the higher PI-RADS categories, however, PI-RADS category 3 showed the lowest level of confidence (median = 6.67, IQR = (6.33–7)).

The level of confidence in rating the different zones of the prostate showed no significant difference between lesions in the transitional and peripheral zones (*p* = 0.9). On the other hand, confidence ratings were higher when the rating was correct compared to when it was incorrect (median = 8.33 and 6.83, respectively, *p* < 0.001), Figure 4b,c show the level of confidence across the zones and correctness of the rating.

### 3.6. Sensitivity Analysis

A sensitivity analysis was conducted to test for the potential influence of finasteride on total PSA and PSA-D. The median total PSA was 10.75 (7.03–25.9) ng/mL, the median prostate volume was 50 (40–75.1) mL, and the median PSA-D was 0.2 (0.11–0.53) ng/mL^2^. The exclusion of patients on finasteride showed no noteworthy changes in the analysis as can be seen in Supplementary Table S3.

## 4. Discussion

In this cohort of 102 patients who underwent TRUS-guided biopsy following mpMRI, we evaluated the inter-reader agreement and diagnostic accuracy of the PI-RADS v2.1 scoring system among experienced radiologists at a tertiary academic center. Overall, inter-reader agreement was substantial, and PI-RADS v2.1 demonstrated good overall diagnostic performance for the detection of PCa as well as csPCa.

International consensus statements endorse the routine use of mpMRI as a triage tool to guide prostate biopsy decisions [17,18]; however, inter-reader variability remains a recognized limitation of mpMRI interpretation. PI-RADS v2.1 was introduced to improve standardization and consistency among readers [8]. In this study, agreement among the three experienced radiologists was moderate to good, with comparable findings across ICC (0.749) and kappa (0.748) analyses. These findings are consistent with those of Brembilla et al., who reported substantial agreement among dedicated readers (κ = 0.621, 95% CI: 0.548–0.694) [19]. Urase et al. and Tamada et al. reported moderate inter-reader agreement among experienced readers using PI-RADS v2.1 with kappa values of 0.509 and 0.645, respectively [20,21]. In contrast, M. Hötker et al. reported only fair agreement, while S. Smani et al. found poor agreement with considerable inter-reader variability across both academic and community care cohorts [22,23].

The slightly higher level of agreement observed in our study may be explained by the predominance of lesions categorized as PI-RADS 4–5, given that inclusion was limited to mpMRI cases with corresponding TRUS-guided biopsies, where agreement tends to be higher. This may also be partially attributed to the readers’ level of dedication and experience. Notably, three experienced readers from different institutions independently evaluated images from a single institution under standardized conditions using the same software platform. This design aimed to reduce the influence of institution-specific practices on inter-reader agreement, indicating that the high level of agreement observed is more likely attributable to reader expertise rather than shared institutional protocols.

The diagnostic performance of PI-RADS v2.1 showed a sensitivity of 100% and specificity of 47.6% for PI-RADS 3–5 in detecting any PCa, and a sensitivity of 100% and specificity of 35.7% for detecting csPCa (defined as Gleason grade group ≥ 2; Gleason score ≥ 3 + 4). The AUC for detection of csPCa was 0.679 based on the average score across the three raters. S. Smani et al. reported comparable diagnostic performance, with no significant difference in AUC between community (AUC = 0.759; 95% CI: 0.702–0.816) and academic (AUC = 0.785; 95% CI: 0.731–0.838) interpreters. The reported PPV of PI-RADS ≥ 3 for csPCa was 0.467 in the community cohort and 0.435 in the academic cohort, while the NPV of PI-RADS ≤ 2 for excluding csPCa was 0.826 and 0.830, respectively [22]. The multicenter PROMIS study reported a sensitivity of 93% and specificity of 41% for mpMRI in detecting csPCa, with an NPV of 89% and a PPV of 51%. Notably, lesions in this study were assessed using the Likert scale rather than PI-RADS; nevertheless, comparative studies have shown comparable performance between the Likert and PI-RADS reporting systems [24].

Similarly, a 2024 systematic review and meta-analysis by Oerther et al. of 70 studies evaluating PI-RADS v2.1 reported that a threshold of PI-RADS ≥ 3 yielded a sensitivity of 96% and a specificity of 43% for csPCa [25].

Moreover, data from a multicenter study demonstrated substantial inter-center variability in the PPV of PI-RADS v2.1, with pooled PPVs of 35% for PI-RADS ≥3 and 49% for PI-RADS ≥ 4 in detecting csPCa [26]. A Cochrane review reported a pooled sensitivity of 91% and specificity of 37% for detecting csPCa (GG ≥ 2), while the corresponding NPV and PPV were 0.91 and 0.88, respectively [27].

PCa most commonly arises in the PZ, with fewer than 30% of cases originating in the TZ [28]. Despite the paucity of studies specifically evaluating TZ lesions, available evidence has generally demonstrated higher inter-reader agreement and diagnostic performance in the PZ compared to the TZ, largely due to the ambiguous imaging features of these lesions [9,29]. PI-RADS v2.1 aimed to address these limitations by introducing more objective criteria for TZ lesion evaluation [8]. Contrary to prior reports, our results showed higher agreement for lesions in the TZ compared to the PZ. This finding may reflect improved standardization of TZ lesion assessment with PI-RADS v2.1, as reflected by higher specificity in the TZ, whereas the PZ maintained higher sensitivity for detecting both any PCa and csPCa across all raters.

Emerging evidence suggests a modest improvement in TZ lesion assessment with PI-RADS v2.1. For instance, Lim et al. supported the utility of the PI-RADS v2.1 upgrade rule for atypical TZ nodules [30]. Similarly, Wei et al. demonstrated that PI-RADS v2.1 achieved better inter-reader agreement and higher diagnostic accuracy (AUC 0.866 vs. 0.827 and 0.929 vs. 0.899) compared to PI-RADS v2 for both TZ cancer detection and csPCa diagnosis [29]. Song et al. reported moderate inter-reader agreement with PI-RADS v2.1, with higher agreement for PZ than TZ lesions [31]. Conversely, Bhayana et al. reported improved inter-reader agreement and diagnostic performance with PI-RADS v2.1 in the PZ but not in the TZ among experienced readers [32]. A systematic review and meta-analysis by Wen et al. showed substantial inter-reader agreement for PI-RADS v2.1 in both whole-gland and TZ lesions, with no significant improvement over PI-RADS v2 [33].

Nevertheless, the higher level of agreement in TZ lesions observed in our study may in part be explained by the relatively smaller number of TZ lesions compared to PZ lesions (35 vs. 67), although this distribution is consistent with the known prevalence of these lesions [28]. Additionally, and as noted by session moderators, the inherent ambiguity of TZ lesions appeared to prompt raters to rely more heavily on the PI-RADS v2.1 atlas for lesion characterization, thereby reducing dependence on individual experience and greater adherence to standardized definitions. Unfortunately, referral to the atlas was not objectively recorded or quantified in this study. These lesions are known to have greater inter-reader variability and often require closer reliance on PI-RADS criteria, particularly in uncertain cases [9].

PSA-D has been shown to be a strong predictor of csPCa, reflecting increased tumor burden relative to prostate volume and correlating with higher Gleason scores and more aggressive disease [34,35].

In this study, PSA-D stratification demonstrated comparable sensitivities across the two PSA-D groups, with sensitivity reaching 100% in both strata. Specificity was relatively low in both groups, and although a trend toward higher specificity in the lower PSA-D group was observed, the wide confidence intervals make it difficult to draw firm conclusions from these estimates. NPV was also 100% in both groups; however, these findings should be interpreted cautiously, particularly in the low PSA-D subgroup, which included only five csPCa cases. In contrast, PPV was substantially higher in the elevated PSA-D group. Although this difference is clinically plausible, it should be interpreted in the context of the markedly higher disease prevalence in the elevated PSA-D subgroup, as PPV is inherently prevalence-dependent. These results suggest that PSA-D may modify the post-test probability associated with a given PI-RADS score, but they do not establish an independent incremental diagnostic benefit beyond PI-RADS alone.

Previous evidence supports incorporating PSA-D into PI-RADS-based risk assessment to improve diagnostic stratification. For instance, Distler et al. demonstrated that incorporating PSA-D improved diagnostic performance, with a higher AUC than PI-RADS alone (0.79 vs. 0.75, *p* < 0.001), with the NPV for csPCa in patients with negative MRI findings increasing from 79% to 89% when PSA-D was ≤0.15 ng/mL^2^ and from 83% to 93% in the repeat biopsy setting, while the NPV for high-grade PCa improved from 92% to 98% in the overall cohort [34]. Hansen et al. found that men with prior negative or low-grade prostate biopsy and low PSA-D (≤0.2 ng/mL^2^) have a low risk of csPCa, favoring surveillance even with mildly suspicious MRI findings [35]. Li et al. showed that combining PI-RADS v2.1 with PSA-D can guide biopsy decisions, safely reducing unnecessary biopsies in selected patients while maintaining detection of csPCa [13]. It should be noted, however, that the low PSA-D subgroup in this study contained only a small number of csPCa events, and these findings should therefore be considered hypothesis-generating rather than confirmatory, pending validation in larger prospective cohorts.

On mpMRI, a typical focal PCa appears as an area of restricted diffusion, low T2-weighted signal intensity, and hyperenhancement on contrast imaging [36]. In this cohort, mpMRI features including DWI hyperintensity, ADC hypointensity, hyperenhancement, and T2 hypointensity were frequently observed in patients with prostate cancer, and all four features were present in every patient with PCa but were less common in those without cancer. In univariate analysis, DWI hyperintensity, ADC hypointensity, and hyperenhancement were significantly associated with PCa and csPCa while only DWI hyperintensity remained independently associated with biopsy-detected PCa and csPCa in multivariable patient-level analyses. Lower ADC values derived from DWI correlate with higher GS and clinical risk, highlighting the value of these metrics as indicators of PCa aggressiveness [11,37]. In line with this, Shigemura et al. reported that positive DWI findings were significantly associated with higher Gleason grades, more frequent positive surgical margins in prostatectomy specimens, and adverse pathological features [12]. Taken together, these findings support an association between DWI hyperintensity of the reader-selected index lesion and patient-level detection of PCa and csPCa. However, given the absence of lesion-level radiologic–pathologic correlation, this finding should be interpreted as a patient-level association rather than evidence that DWI hyperintensity independently predicts malignancy within the corresponding lesion.

Raters reported consistently high confidence across PI-RADS categories, except for PI-RADS 3, which showed the lowest median confidence. This is clinically relevant, as PI-RADS 3 is the most diagnostically uncertain category [8,9] and the setting in which PSA-D is particularly useful for guiding biopsy decisions [13]. The higher confidence observed in correct compared with incorrect ratings suggests that confidence may serve as a qualitative indicator of diagnostic certainty, although this requires prospective validation.

This study has several limitations. It is a single-center retrospective study with a relatively small sample size. Inclusion was restricted to patients with a corresponding TRUS-guided biopsy, resulting in a biopsy-enriched cohort with a cancer prevalence of 58.8%. This is higher than that seen in unselected screening populations and led to a relative paucity of PI-RADS 1–2 cases, which limits generalizability to routine clinical practice.

All raters had access to the official PI-RADS v2.1 instructional atlas during the rating sessions; however, the frequency and extent of atlas consultation were not recorded. We therefore cannot determine whether atlas use contributed to the higher agreement observed for transition-zone lesions. This may limit the generalizability of the zone-specific findings to settings with different access to, or reliance on, the atlas; accordingly, the higher transition-zone agreement should be interpreted as hypothesis-generating.

A further limitation is that, although cognitively targeted cores were obtained from the mpMRI-identified index lesion in addition to systematic TRUS-guided biopsy, MRI–ultrasound fusion biopsy was unavailable. Cognitive targeting is less precise than fusion-based and may have limited lesion-level radiologic-pathologic correlation [5]. Lesions demonstrating imaging features newly described in PI-RADS v2.1, particularly those of the TZ, were not specifically assessed, partly due to the rarity of such cases.

Finally, lesion-level spatial concordance between the reader-selected index lesion and the biopsy-positive site was not established. Therefore, associations between index-lesion MRI features and biopsy-detected PCa or csPCa represent patient-level associations and should not be interpreted as definitive lesion-level radiologic-pathologic correlations.

In conclusion, this study provides a holistic evaluation of PI-RADS v2.1 in a real-world clinical setting, demonstrating reliable inter-reader agreement and good diagnostic performance for PCa. DWI hyperintensity was the most consistent predictor of both PCa and csPCa. Inter-reader agreement was substantial and higher in TZ compared to PZ lesions. Raters also demonstrated consistently high confidence overall, with lower confidence in PI-RADS 3, reflecting its inherent diagnostic ambiguity. Future prospective studies with larger sample sizes, enhanced training in the PI-RADS system, and evaluation of the role of AI in lesion interpretation are warranted.

## Figures and Tables

**Table 1:** Characteristics of the study sample.

Variable	n = 102
Age	66 (60.75–73)
Total PSA, ng/mL	10.54 (7.03–22.33)
Prostate volume, mL	58 (41.75–81.75)
PSA density, ng/mL^2^	0.17 (0.1–0.43)
Finasteride use	16 (15.7%)
Histopathology on TRUS biopsy	
Normal histology	28 (27.5)
BPH	14 (13.7)
Prostatitis	6 (5.9)
PIN	4 (3.9)
Adenocarcinoma	60 (58.8)
Grade group 1	14 (23.3)
Grade group 2	16 (26.7)
Grade group 3	7 (11.7)
Grade group 4	13 (21.7)
Grade group 5	10 (16.7)

Median (IQR), n (%), PSA: Prostate specific antigen, BPH: Benign prostatic hyperplasia, PIN: Prostate intraepithelial neoplasia, Samples can show multiple histological features.

**Table 2:** PI-RADS scoring results for each rater.

PI-RADS	Rater 1	Rater 2	Rater 3
Median (IQR)	4 (3–5)	4 (3–5)	4 (2–5)
PI-RADS 1 & 2	11 (10.8%)	19 (18.6%)	26 (25.5%)
PI-RADS 3	19 (18.6%)	11 (10.8%)	7 (6.9%)
PI-RADS 4 & 5	72 (70.6%)	72 (70.6%)	69 (67.6%)

**Table 3:** Diagnostic performance of the three raters using the PI-RADS v2.1 scoring system for detecting cancer and clinically significant cancer.

Rater	Zone	PCa		csPCa	
		Sensitivity	Specificity	Sensitivity	Specificity
Rater 1	Overall	100% (94–100%)	26.2% (13.9–42%)	100% (92.3–100%)	19.6% (10.2–32.4%)
	Transitional	100.0% (78.2–100.0%)	52.6% (28.9–75.6%)	100.0% (76.8–100.0%)	50% (27.2–72.8%)
	Peripheral	100.0% (92.1–100.0%)	4.3% (0.1–21.9%)	100.0% (89.1–100.0%)	2.8% (0.1–14.5%)
Rater 2	Overall	96.7% (88.5–99.6%)	40.5% (25.6–56.7%)	95.7% (85.2–99.5%)	30.4% (18.8–44.1%)
	Transitional	86.7% (59.5–98.3%)	63.2% (38.4–83.7%)	85.7% (57.2–98.2%)	60.0% (36.1–80.9%)
	Peripheral	100.0% (92.1–100.0%)	21.7% (7.5–43.7%)	100.0% (89.1–100.0%)	13.9% (4.7–29.5%)
Rater 3	Overall	95% (86.1–99%)	54.8% (38.7–70.2%)	100% (92.3–100%)	46.4% (33–60.3%)
	Transitional	100.0% (78.2–100.0%)	73.7% (48.8–90.9%)	100.0% (76.8–100.0%)	70.0% (45.7–88.1%)
	Peripheral	93.3% (81.7–98.6%)	39.1% (19.7–61.5%)	100.0% (89.1–100.0%)	33.3% (18.6–51%)
Average	Overall	100% (94–100%)	47.6% (32–63.6%)	100% (92.3–100%)	35.7% (23.4–49.6%)
	Transitional	100.0% (78.2–100.0%)	73.7% (48.8–90.9%)	100.0% (76.8–100.0%)	70.0% (45.7–88.1%)
	Peripheral	100.0% (92.1–100.0%)	26.1% (10.2–48.4%)	100.0% (89.1–100.0%)	16.7% (6.4–32.8%)

Mean (95% CI), PI-RADS score ≥ 3 was considered suggestive of cancer, csPCa: clinically significant prostate cancer.

**Table 4:** Diagnostic performance of the three raters using the PI-RADS v2.1 scoring system for detecting cancer and clinically significant cancer between two risk groups stratified according to PSA density.

PSA Density	Overall, *N*	PCa, *n* (%)	csPCa, *n* (%)	PCa				csPCa			
				Sensitivity	Specificity	NPV	PPV	Sensitivity	Specificity	NPV	PPV
<0.15 ng/mL^2^	44	13 (29.5%)	5 (11.4%)	100.0% (75.3–100.0%)	51.6% (33.1–69.8%)	100.0% (79.4–100.0%)	46.4% (27.5–66.1%)	100.0% (47.8–100.0%)	41% (25.6–57.9%)	100.0% (79.4–100.0%)	17.9% (6.1–36.9%)
≥0.15 ng/mL^2^	58	47 (81%)	41 (70.7%)	100.0% (92.5–100.0%)	36.4% (10.9–69.2%)	100.0% (39.8–100.0%)	87% (75.1–94.6%)	100.0% (91.4–100.0%)	23.5% (6.8–49.9%)	100.0% (39.8–100.0%)	75.9% (62.4–86.5%)

Estimate (95% CI), positive PI-RADS score was ≥3; PSA, prostate-specific antigen; PCa, prostate cancer; csPCa, clinically significant prostate cancer; PPV, positive predictive value; NPV, negative predictive value.

**Table 5:** Patient-level associations between MRI features of the reader-selected index lesion and biopsy-detected PCa and csPCa.

Variable	Univariate Model				Multivariate Model			
	PCa		csPCa		Pca		csPCa	
	LogOR (95%CI)	*p*-Value	LogOR (95%CI)	*p*-Value	LogOR (95%CI)	*p*-Value	LogOR (95%CI)	*p*-Value
DWI Hyperintensity	4.01 (1.94–8.88)	<0.001	3.36 (1.29–8.23)	<0.001	3.23 (1.01–8.12)	0.002	2.52 (0.34–7.39)	0.020
ADC Hypointensity	3.54 (1.42–8.41)	<0.001	2.92 (0.81–7.79)	0.003	2.43 (−0.14–7.38)	0.066	1.72 (−0.78–6.65)	0.193
Hyperenhancement	3.07 (0.89–7.95)	0.002	2.48 (0.30–7.36)	0.021	2.45 (−0.14–7.40)	0.065	1.73 (−0.80–6.66)	0.197
T2 Hypointensity	2.01 (−0.53–6.95)	0.128	1.45 (−1.09–6.39)	0.288	-	-	-	-

Pca: Prostate cancer, csPCa: clinically significant prostate cancer, LogOR: Log odds ratio, 95%CI: 95% confidence interval, DWI: Diffusion weighted imaging, ADC: Apparent diffusion coefficient.

## Data Availability

The data presented in this study are available upon reasonable request from the corresponding author. The data are not publicly available because they contain potentially identifiable patient information and are subject to privacy and ethical restrictions.

## Supplementary Materials

The following supporting information can be downloaded at: https://www.mdpi.com/article/10.3390/diagnostics16183056/s1, Table S1. PI-RADS 3 stratification according to PSA density; Table S2. Frequency of selected radiological findings on mpMRI; Table S3. Sensitivity analysis of the diagnostic performance of the three raters using the PI-RADS v2.1 scoring system for detecting cancer and clinically significant cancer between two risk groups stratified accord-ing to PSA density after excluding patients receiving finasteride; Figure S1. This transition-zone lesion showed complete concordance among the three raters, who all assigned a PI-RADS score of 2, consistent with its benign histopathology. (a) T2-weighted image showing an en-capsulated nodule; (b) DWI showing no focal marked hyperintensity; (c) ADC map demonstrating mildly hy-pointense signal within the nodule; and (d) corresponding histopathology slide showing benign prostatic tissue with glandular and stromal components; Figure S2. This anterior transition-zone lesion showed complete concordance among the three raters, who all assigned a PI-RADS score of 4, consistent with its malignant histopathology. (a) Axial T2-weighted image showing an ill-defined, lenticular, hypointense anterior transition-zone lesion; (b) ADC map demonstrating mild hypointensity within the lesion; and (c) corresponding histopathology slide showing prostatic adenocarcino-ma, Gleason score 7 (Grade Group 2; 3+4), with perineural invasion; Figure S3. This benign transition-zone lesion showed complete discordance among the three raters, with each assigning a different PI-RADS score (2, 3, and 5, respectively). (a) T2-weighted image showing a transi-tion-zone lesion; (b) high b-value DWI showing restricted diffusion; (c) ADC map demonstrating the same lesion; and (d) corresponding histopathology slide showing benign prostatic tissue; Figure S4. This benign peripheral-zone lesion showed complete discordance among the three raters, with each assigning a different PI-RADS score (2, 3, and 4, respectively). (a) Axial T2-weighted image showing a wedge-shaped peripheral-zone lesion; (b) coronal T2-weighted image demonstrating the lesion; (c) sagittal T2-weighted image demonstrating the same lesion; (d) ADC map showing the lesion; (e) high b-value DWI show-ing restricted diffusion; and (f) corresponding histopathology slide showing benign prostatic tissue.

## Abbreviations

PCa	Prostate cancer
mpMRI	Multiparametric magnetic resonance imaging
PI-RADS	Prostate Imaging Reporting and Data System
PSA-D	Prostate-specific antigen density
TRUS	Transrectal ultrasound
ICC	Intraclass correlation coefficient
AUC	Area under the receiver operating characteristic curve
TZ	Transition zone
PZ	Peripheral zone
csPCa	Clinically significant prostate cancer
PPV	Positive predictive value
DWI	Diffusion-weighted imaging
PSA	Prostate-specific antigen
MRI	Magnetic resonance imaging
T2WI	T2-weighted imaging
ADC	Apparent diffusion coefficient
DCE	Dynamic contrast-enhanced imaging
ISUP	International Society of Urological Pathology
IQR	Interquartile range
CI	Confidence interval
NPV	Negative predictive value
BPH	Benign prostatic hyperplasia
PIN	Prostatic intraepithelial neoplasia
GG	Grade group

## References

[1] Siegel R.L., Kratzer T.B., Giaquinto A.N., Sung H., Jemal A. (2025). Cancer statistics, 2025. CA Cancer J. Clin..

[2] Descotes J.L. (2019). Diagnosis of prostate cancer. Asian J. Urol..

[3] Heijnsdijk E.A.M., Der Kinderen A., Wever E.M., Draisma G., Roobol M.J., De Koning H.J. (2009). Overdetection, overtreatment and costs in prostate-specific antigen screening for prostate cancer. Br. J. Cancer.

[4] Turkbey B., Brown A.M., Sankineni S., Wood B.J., Pinto P.A., Choyke P.L. (2016). Multiparametric prostate magnetic resonance imaging in the evaluation of prostate cancer. CA Cancer J. Clin..

[5] Bjurlin M.A., Mendhiratta N., Wysock J.S., Taneja S.S. (2016). Multiparametric MRI and targeted prostate biopsy: Improvements in cancer detection, localization, and risk assessment. Cent. Eur. J. Urol..

[6] Sonn G.A., Fan R.E., Ghanouni P., Wang N.N., Brooks J.D., Loening A.M., Daniel B.L., To’o K.J., Thong A.E., Leppert J.T. (2019). Prostate Magnetic Resonance Imaging Interpretation Varies Substantially Across Radiologists. Eur. Urol. Focus..

[7] Barentsz J.O., Richenberg J., Clements R., Choyke P., Verma S., Villeirs G., Rouviere O., Logager V., Fütterer J.J. (2012). ESUR prostate MR guidelines 2012. Eur. Radiol..

[8] Turkbey B., Rosenkrantz A.B., Haider M.A., Padhani A.R., Villeirs G., Macura K.J., Tempany C.M., Choyke P.L., Cornud F., Margolis D.J. (2019). Prostate Imaging Reporting and Data System Version 2.1: 2019 Update of Prostate Imaging Reporting and Data System Version 2. Eur. Urol..

[9] Rosenkrantz A.B., Ginocchio L.A., Cornfeld D., Froemming A.T., Gupta R.T., Turkbey B., Westphalen A.C., Babb J.S., Margolis D.J. (2016). Interobserver Reproducibility of the PI-RADS Version 2 Lexicon: A Multicenter Study of Six Experienced Prostate Radiologists. Radiology.

[10] Rudolph M.M., Baur A.D.J., Cash H., Haas M., Mahjoub S., Hartenstein A., Hamm C.A., Beetz N.L., Konietschke F., Hamm B. (2020). Diagnostic performance of PI-RADS version 2.1 compared to version 2.0 for detection of peripheral and transition zone prostate cancer. Sci. Rep..

[11] Turkbey B., Shah V.P., Pang Y., Bernardo M., Xu S., Kruecker J., Locklin J., Baccala A.A., Rastinehad A.R., Merino M.J. (2011). Is Apparent Diffusion Coefficient Associated with Clinical Risk Scores for Prostate Cancers that Are Visible on 3-T MR Images?. Radiology.

[12] Shigemura K., Yamanaka N., Yamashita M. (2013). Can diffusion-weighted magnetic resonance imaging predict a high Gleason score of prostate cancer?. Korean J. Urol..

[13] Li Y., Wang S., Wang J., Qi X., Liu T., He X., Zhang Y., Zhu Y., Zeng Y. (2025). PI-RADSv2.1 combined with PSA density for optimizing prostate biopsy decisions: A retrospective analysis. Front. Oncol..

[14] Epstein J.I., Egevad L., Amin M.B., Delahunt B., Srigley J.R., Humphrey P.A. (2016). The 2014 International Society of Urological Pathology (ISUP) Consensus Conference on Gleason Grading of Prostatic Carcinoma: Definition of Grading Patterns and Proposal for a New Grading System. Am. J. Surg. Pathol..

[15] R Core Team (2024). R: A Language and Environment for Statistical Computing.

[16] Koo T.K., Li M.Y. (2016). A Guideline of Selecting and Reporting Intraclass Correlation Coefficients for Reliability Research. J. Chiropr. Med..

[17] Carter H.B., Albertsen P.C., Barry M.J., Etzioni R., Freedland S.J., Greene K.L., Holmberg L., Kantoff P., Konety B.R., Murad M.H. (2013). Early Detection of Prostate Cancer: AUA Guideline. J. Urol..

[18] Cornford P., Van Den Bergh R.C.N., Briers E., Van Den Broeck T., Brunckhorst O., Darraugh J., Eberli D., De Meerleer G., De Santis M., Farolfi A. (2024). EAU-EANM-ESTRO-ESUR-ISUP-SIOG Guidelines on Prostate Cancer—2024 Update. Part I: Screening, Diagnosis, and Local Treatment with Curative Intent. Eur. Urol..

[19] Brembilla G., Dell’Oglio P., Stabile A., Damascelli A., Brunetti L., Ravelli S., Cristel G., Schiani E., Venturini E., Grippaldi D. (2020). Interreader variability in prostate MRI reporting using Prostate Imaging Reporting and Data System version 2.1. Eur. Radiol..

[20] Tamada T., Kido A., Takeuchi M., Yamamoto A., Miyaji Y., Kanomata N., Sone T. (2019). Comparison of PI-RADS version 2 and PI-RADS version 2.1 for the detection of transition zone prostate cancer. Eur. J. Radiol..

[21] Urase Y., Ueno Y., Tamada T., Sofue K., Takahashi S., Hinata N., Harada K., Fujisawa M., Murakami T. (2022). Comparison of prostate imaging reporting and data system v2.1 and 2 in transition and peripheral zones: Evaluation of interreader agreement and diagnostic performance in detecting clinically significant prostate cancer. Br. J. Radiol..

[22] Smani S., Jalfon M., Sundaresan V., Lokeshwar S.D., Nguyen J., Halstuch D., Khajir G., Cavallo J.A., Sprenkle P.C., Leapman M.S. (2025). Inter-reader reliability and diagnostic accuracy of PI-RADS scoring between academic and community care networks: How wide is the gap?. Urol. Oncol..

[23] Hötker A.M., Blüthgen C., Rupp N.J., Schneider A.F., Eberli D., Donati O.F. (2020). Comparison of the PI-RADS 2.1 scoring system to PI-RADS 2.0: Impact on diagnostic accuracy and inter-reader agreement. PLoS ONE.

[24] Ahmed H.U., El-Shater Bosaily A., Brown L.C., Gabe R., Kaplan R., Parmar M.K., Collaco-Moraes Y., Ward K., Hindley R.G., Freeman A. (2017). Diagnostic accuracy of multi-parametric MRI and TRUS biopsy in prostate cancer (PROMIS): A paired validating confirmatory study. Lancet.

[25] Oerther B., Nedelcu A., Engel H., Schmucker C., Schwarzer G., Brugger T., Schoots I.G., Eisenblaetter M., Sigle A., Gratzke C. (2024). Update on PI-RADS Version 2.1 Diagnostic Performance Benchmarks for Prostate MRI: Systematic Review and Meta-Analysis. Radiology.

[26] Westphalen A.C., McCulloch C.E., Anaokar J.M., Arora S., Barashi N.S., Barentsz J.O., Bathala T.K., Bittencourt L.K., Booker M.T., Braxton V.G. (2020). Variability of the Positive Predictive Value of PI-RADS for Prostate MRI across 26 Centers: Experience of the Society of Abdominal Radiology Prostate Cancer Disease-focused Panel. Radiology.

[27] Drost F.J.H., Osses D.F., Nieboer D., Steyerberg E.W., Bangma C.H., Roobol M.J., Schoots I.G. (2019). Prostate MRI, with or without MRI-targeted biopsy, and systematic biopsy for detecting prostate cancer. Cochrane Database Syst. Rev..

[28] McNeal J.E., Redwine E.A., Freiha F.S., Stamey T.A. (1988). Zonal Distribution of Prostatic Adenocarcinoma: Correlation with Histologic Pattern and Direction of Spread. Am. J. Surg. Pathol..

[29] Wei C., Zhang Y., Pan P., Chen T., Yu H., Dai G., Tu J., Yang S., Zhao W., Shen J. (2021). Diagnostic Accuracy and Interobserver Agreement of PI-RADS Version 2 and Version 2.1 for the Detection of Transition Zone Prostate Cancers. Am. J. Roentgenol..

[30] Lim C.S., Abreu-Gomez J., Carrion I., Schieda N. (2021). Prevalence of Prostate Cancer in PI-RADS Version 2.1 Transition Zone Atypical Nodules Upgraded by Abnormal DWI: Correlation With MRI-Directed TRUS-Guided Targeted Biopsy. Am. J. Roentgenol..

[31] Song W.H., Kim T.U., Ryu H.S., Yun M.S., Park S.W. (2025). Enhancement of inter-/intra-reader agreement using the Prostate Imaging Reporting and Data System version 2.1 for prostate cancer detection in magnetic resonance imaging/transrectal ultrasound software fusion prostate biopsy. Investig. Clin. Urol..

[32] Bhayana R., O’Shea A., Anderson M.A., Bradley W.R., Gottumukkala R.V., Mojtahed A., Pierce T.T., Harisinghani M. (2021). PI-RADS Versions 2 and 2.1: Interobserver Agreement and Diagnostic Performance in Peripheral and Transition Zone Lesions Among Six Radiologists. Am. J. Roentgenol..

[33] Wen J., Ji Y., Han J., Shen X., Qiu Y. (2022). Inter-reader agreement of the prostate imaging reporting and data system version v2.1 for detection of prostate cancer: A systematic review and meta-analysis. Front. Oncol..

[34] Distler F.A., Radtke J.P., Bonekamp D., Kesch C., Schlemmer H.P., Wieczorek K., Kirchner M., Pahernik S., Hohenfellner M., Hadaschik B.A. (2017). The Value of PSA Density in Combination with PI-RADS^TM^ for the Accuracy of Prostate Cancer Prediction. J. Urol..

[35] Hansen N.L., Barrett T., Koo B., Doble A., Gnanapragasam V., Warren A., Kastner C., Bratt O. (2017). The influence of prostate-specific antigen density on positive and negative predictive values of multiparametric magnetic resonance imaging to detect Gleason score 7–10 prostate cancer in a repeat biopsy setting. BJU Int..

[36] Spektor M., Mathur M., Weinreb J.C. (2017). Standards for MRI reporting-the evolution to PI-RADS v 2.0. Transl. Androl. Urol..

[37] Stabile A., Giganti F., Rosenkrantz A.B., Taneja S.S., Villeirs G., Gill I.S., Allen C., Emberton M., Moore C.M., Kasivisvanathan V. (2020). Multiparametric MRI for prostate cancer diagnosis: Current status and future directions. Nat. Rev. Urol..

