# Supplementary material for: An Assessment of PI-RADS v2.1: Inter-Reader Agreement, Diagnostic Accuracy, and the Added Value of PSA Density in Prostate Cancer Prediction
Source: Diagnostics (Basel). 2026 Sep 20;16(18):3056. doi: 10.3390/diagnostics16183056 (PMC13605971; doi:10.3390/diagnostics16183056)
Supplement: Supplementary file 1 [file diagnostics-16-03056-s001.zip › diagnostics-4548711-supplementary.pdf]

Supplementary Tables

Supplementary Table S1

PI-RADS 3 stratification according to PSA density

PI-RADS	PSA density	Rater 1			Rater 2			Rater 3		
		n	Pca	csPca	n	Pca	csPca	n	Pca	csPca
PI-RADS 3	Total	19	4 (21.1%)	4 (21.1%)	11	1 (9.1%)	0 (0%)	7	1 (14.3%)	0 (0%)
	<0.15 ng/mL^2	14	2 (14.3%)	2 (14.3%)	9	1 (11.1%)	0 (0%)	7	1 (14.3%)	0 (0%)
	≥0.15 ng/mL^2	5	2 (40%)	2 (40%)	2	0 (0%)	0 (0%)	0	0 (0%)	0 (0%)

Supplementary Table S2

Frequency of certain radiological findings on mpMRI

Finding	**Total,** *n = 102*	**PCa,** *n = 60*
DWI Hyperintensity	89 (87.3%)	60 (100%)
ADC Hypointensity	93 (91.2%)	60 (100%)
Hyperenhancement	96 (94.1%)	60 (100%)
T2 Hypointensity	100 (98%)	60 (100%)

n (%), mpMRI: Multiparametric MRI

Supplementary Table S3

Sensitivity analysis for the diagnostic performance of the three raters using the PI-RADS v2.1 scoring system for detecting cancer and clinically significant cancer between two risk groups stratified according to PSA density after excluding patients on Finasteride

PSA density	Overall, *N*	PCa, *n (%)*	csPCa, *n (%)*	Cancer		csCancer	
				Sensitivity	Specificity	Sensitivity	Specificity
<0.15 ng/mL^2	32	12 (37.5%)	5 (15.6%)	100.0% (73.5%-100.0%)	45% (23.1%-68.5%)	100.0% (47.8%-100.0%)	33.3% (16.5%-54%)
≥0.15 ng/mL^2	54	45 (83.3%)	39 (72.2%)	100.0% (92.1%-100.0%)	22.2% (2.8%-60%)	100.0% (91%-100.0%)	13.3% (1.7%-40.5.%)

Supplementary Figures

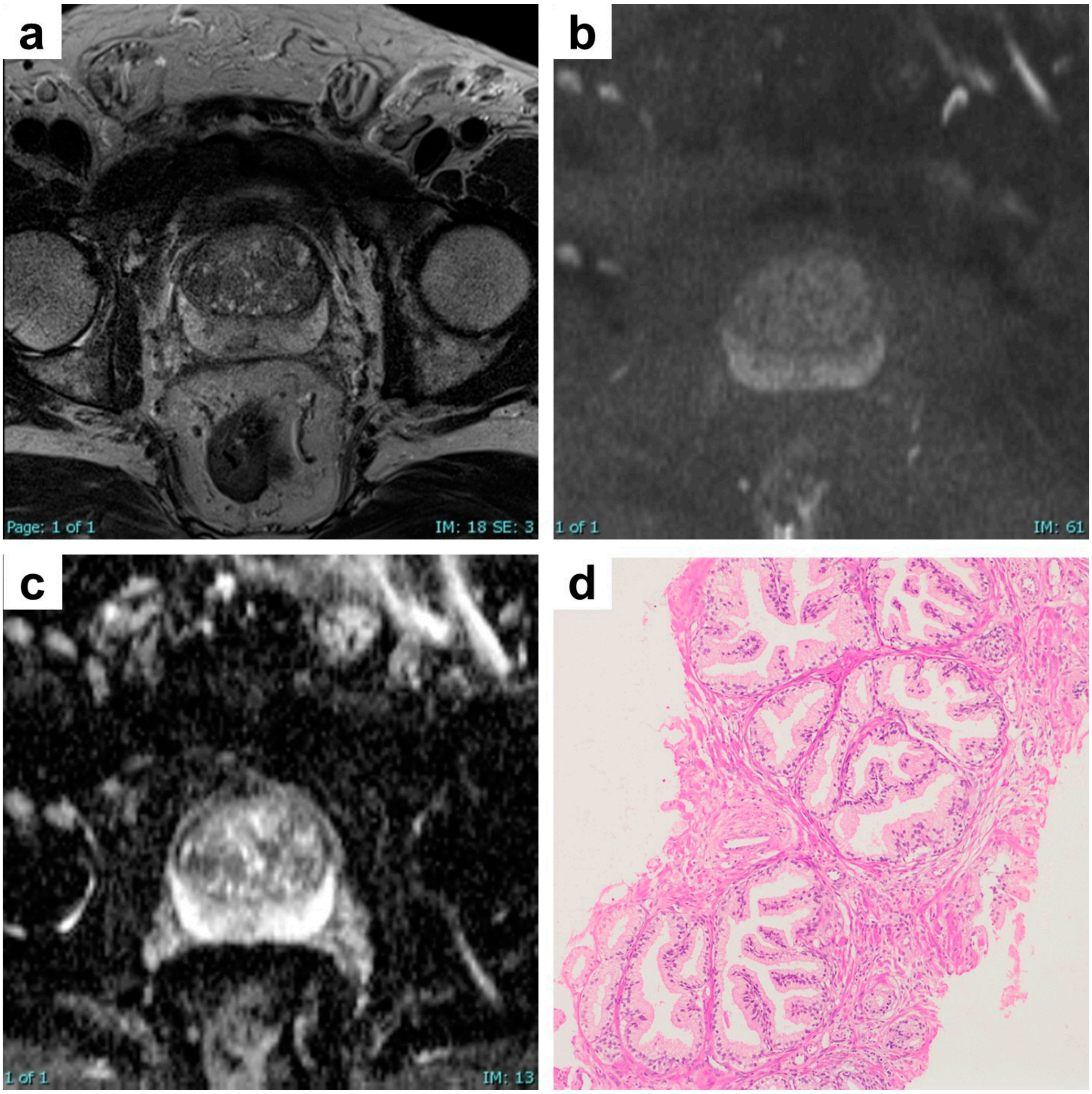

Supplementary Figure S1. This transition-zone lesion showed complete concordance between the three raters where they all scored it as PI-RADS = 2, which aligned with its benign histopathology. a) T2-weighted image showing an encapsulated nodule, b) DWI showing no focal marked hyperintensity, c) ADC map demonstrating mildly hypointense signal within the nodule, and d) the corresponding histopathology slide showing benign prostatic tissue with glandular and stromal components

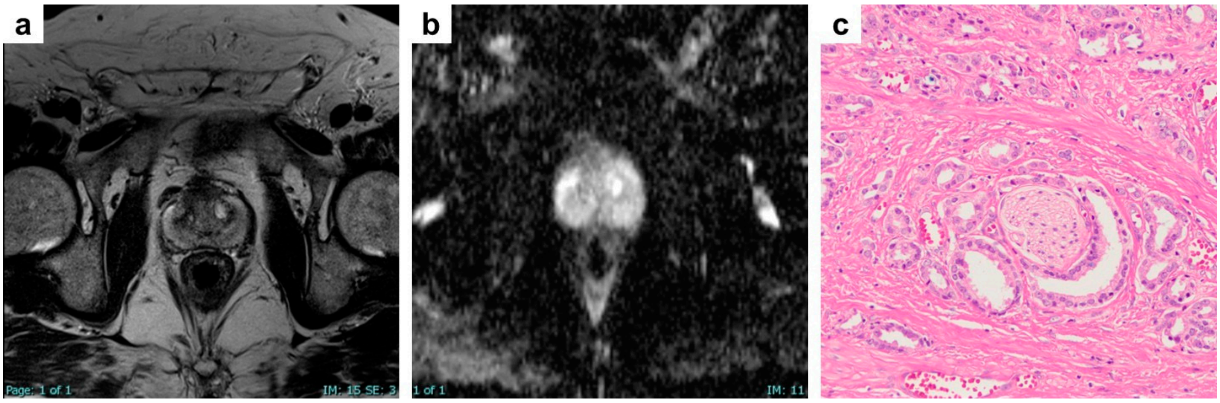

Supplementary Figure S2. This anterior transition-zone lesion showed complete concordance between the three raters where they all scored it as PI-RADS = 4, which aligned with its malignant histopathology. a) axial T2-weighted image showing an ill-defined, lenticular, hypointense, anterior transition-zone lesion, b) ADC map demonstrating mild hypointensity within the lesion, and c) the corresponding histopathology slide showing prostatic adenocarcinoma, Gleason score 7 (Grade group 2; 3+4), with perineural invasion

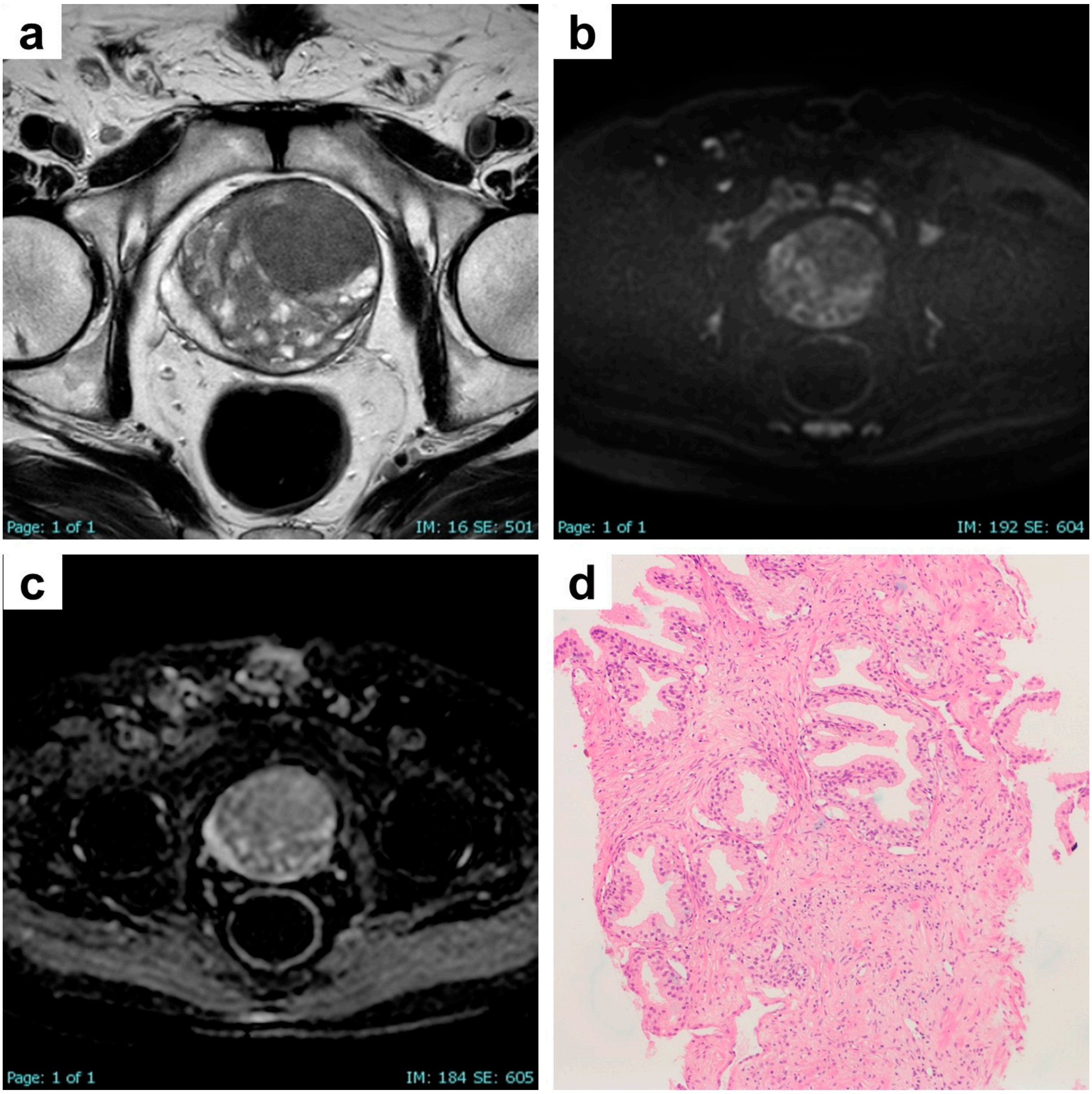

Supplementary Figure S3. This benign transition-zone lesion showed complete discordance between the three raters where each one assigned a different PI-RADS score (2, 3, and 5, respectively). a) T2-weighted image showing a transition-zone lesion, b) high b-value DWI showing restricted diffusion, c) ADC map demonstrating the same lesion, and d) the corresponding histopathology slide showing benign prostatic tissue

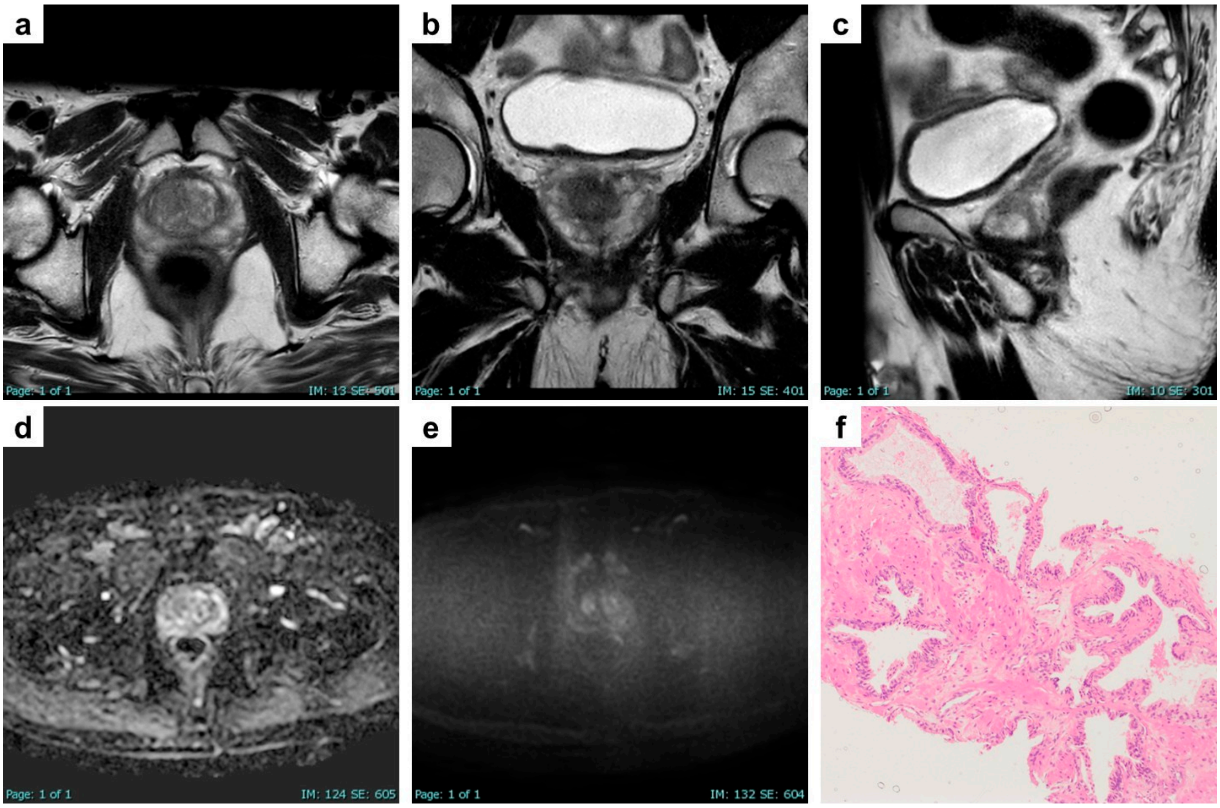

Supplementary Figure S4. This benign peripheral-zone lesion showed complete discordance between the three raters where each one assigned a different PI-RADS score (2, 3, and 4, respectively). a) axial T2-weighted image showing a wedge-shaped peripheral-zone lesion, b) coronal T2-weighted image demonstrating the lesion, c) sagittal T2-weighted image demonstrating the same lesion, d) ADC map showing the lesion, e) high b-value DWI showing restricted diffusion, and f) the corresponding histopathology slide showing benign prostatic tissue
